# Anaphylactic reactions presenting with hypertension

**DOI:** 10.1186/s40064-016-2913-y

**Published:** 2016-08-02

**Authors:** Emrullah Solmazgul, Ali Kutlu, Salim Dogru, Veysel Ozalper, Ibrahim Cetindagli, Ogun Sezer, Musa Salmanoglu, Erol Kilic, Ercan Karabacak, Sami Ozturk

**Affiliations:** 1 Department of Internal Medicine, GATA Haydarpasa Training Hospital, Istanbul, Turkey; 2 Department of Allergy and Immunology, GATA Haydarpasa Training Hospital, Istanbul, Turkey; 3 Department of Otolaryngology, GATA Haydarpasa Training Hospital, Istanbul, Turkey; 4 Department of Internal Medicine, Hakkari Military Hospital, Hakkari, Turkey; 5 Department of Rheumatology, GATA Gulhane Military Medical Academy, Ankara, Turkey; 6 Department of Microbiology and Clinical Microbiology, GATA Haydarpasa Training Hospital, Istanbul, Turkey; 7 Department of Chest Disease, Kasimpasa Military Hospital, Istanbul, Turkey; 8 Department of Dermatology, GATA Haydarpasa Training Hospital, Istanbul, Turkey

**Keywords:** Anaphylactic reactions, Hypertension, Prevalence, Adrenaline

## Abstract

**Background:**

Although a few case reports about hypertensive anaphylaxis (HA) are available in the present literature, there is no study about the prevalence of HA. In this study, we review our cases with anaphylaxis presenting with hypertension and ascertain its prevalence. The documents of the patients who had anaphylactic reactions after the procedures performed for the diagnosis and treatment of allergic diseases in GATA Haydarpasa Clinic of Allergy and Immunology between January 2010 and December 2014 were retrospectively reviewed. Within the study period, 324 patients had undergone 4332 procedures in which 62 of them had developed anaphylaxis.

**Results:**

During the procedures, the rate of anaphylaxis was found to be 1.43 %. The rate of HA among the anaphylaxis patients was 12.9 % (8 of 62 patients). During treatments, 2 patients received adrenaline injections without any adverse reaction.

**Conclusions:**

HA may be seen at a considerable rate during an anaphylactic reaction. Anaphylaxis and hypertension can be recovered by adrenaline injection when required. According to the best of our knowledge, this study is the first original study about the prevalence of HA in English-language medical literature.

## Background

Anaphylaxis is an acute, potentially life-threatening systemic allergic reaction which has a particular importance to all health-care professionals. Diagnosis of anaphylaxis is usually based on the history of allergen exposure and physical findings (Sampson et al. 2006; Simons 2010).

Anaphylactic reactions like airway, breathing and circulatory problems are not always associated with skin and mucosal changes. Other than the upper airway obstruction, cardiovascular collapse is the second most life-threatening anaphylactic reaction which requires an urgent treatment. Reduced blood pressure after exposure is known to be the leading indicator of anaphylactic reaction (Muraro et al. 2014).

Hypotension is the most common cardiovascular system finding and considered to be a criterion for diagnosis of anaphylaxis. Nonetheless, we hypothesize that hypertension can also be encountered in anaphylactic reactions due to compensatory vasopressor responses. Although there are a few case reports in literature, no study about the prevalence of hypertensive anaphylaxis (HA) has been reported. In this study, we analyzed our cases with anaphylaxis who presented with hypertension in order to ascertain the prevalence of HA.

## Methods

The documents of the patients who had anaphylactic reactions after the procedures performed for the diagnosis and treatment of allergic diseases in GATA Haydarpasa Clinic of Allergy and Immunology between January 2010 and December 2014 were retrospectively reviewed. The procedures causing anaphylactic reactions were allergen immunotherapy, allergy provocation tests (medication, food, physical urticaria, exercises) and allergic skin tests.

Before all the procedures, a follow-up form has been created. Information about medical histories of the patients were obtained, vital signs were measured and recorded to the follow-up form. First blood pressure value measured was regarded as the basal blood pressure. Patient’s blood pressure was recorded and evaluated according to the instructions mentioned in Eighth Joint National Committee Guideline (James et al. 2013). Patients who had been previously diagnosed with hypertension, treated for hypertension or measured hypertensive as basal blood pressure were excluded from the study. Patients with any previous diagnosis of multiple drug intolerance syndrome, neuropsychiatric diseases (anxiety, panic disorder, psychogenic dyspnea, dissociative disorder, globus hystericus, conversion) were also excluded from the study. Clinical criteria for diagnosing anaphylaxis were based on The European Academy of Allergy and Clinical Immunology (EAACI) 2014 Guideline (Muraro et al. 2014). Following initial symptoms of anaphylaxis, patients those with the arterial tension of over 140/90 mmHg were considered to have a hypertensive anaphylactic reaction (James et al. 2013).

For patients who were interfered with the diagnosis of hypertensive anaphylactic reaction, the factors causing anaphylactic reaction, symptoms, arterial blood pressure (BP) measurements and the treatments performed were all noted and evaluated. After the symptoms decreased, the patients were kept under observation, vital signs were monitored hourly for 6 h.

The local ethics committee approved the study protocol and written informed consent was obtained from all participants.

## Results

Within the study period, 324 patients had undergone 4332 procedures in which 62 of them had developed anaphylaxis. During these procedures, the rate of anaphylaxis was found to be 1.43 %. Eight patients with HA fitted the criteria mentioned above (4 females, 4 males). The rate of HA among the anaphylaxis patients was 12.9 % (Table 1). The mean age of HA patients was 25.8 years. Among HA patients, two had anaphylactic reaction following bee venom administration (1 patient received vespula venom, 1 patient received *Apis mellifera* therapy), one had anaphylactic reaction following fresh prick test with multi-foods, four had anaphylaxis following house dust mite (HDM) mixture administration, and one had anaphylactic reaction after the application of omalizumab (Table 2). Reactions developed in the course of the increasing dose application in 1 patient who received a bee venom immunotherapy and in 2 patients who received a HDM immunotherapy.

**Table 1:** Patients tested or treated for allergy and prevalence of anaphylaxis

Immunotherapy—test type	Mean age (years)	Number of patients	Number of procedures	Number of total anaphylaxis/procedures (%)	Hypertensive anaphylaxis/total anaphylaxis (%)
Bee-venom	33	25	569	15/569 (2.63)	2/15 (13.33)
Food-drug provocation test	42	36	101	32/101 (3.12)	1/32 (3.12)
Inhalen Allergen IT (immunotherapy)	27.7	177	3194	14/3194 (0.43)	4/14 (28.57)
Drug Injection (Omalizumab)	44	86	468	1/468 (0.21)	1/1 (100)
Total	36	324	4332	62/4332 (1.43)	8/62 (12.90)

**Table 2:** Patients who developed hypertensive anaphylaxis and their managements

Case number	Sex/age (years)	Allergic disease	Causes of anaphylaxis	Additional symptoms^a^	ABP after anaphylaxis^b^ (mmHg)	Treatment
1.	M/20	Bee venom allergy	Venom immunotherapy (vespula 100 mcg)	Papular urticarial lesions	170/100	ivAH, ivSt, nBD
2.	M/20	Bee venom allergy	Venom immunotherapy (*Apis mellifera* 80 mcg)		220/150	ivAH, ivSt, nBD
3.	M/20	Anaphylaxis with multiple food	Prick test with multiple fresh food	Dyspnea, generalize edema	190/120	ivAH, ivSt, nBD
4.	M/37	Allergic rhinitis	Immunotherapy with HDM mix (100.000 SQ-U/ml 0.5 cc)		170/110	ivAH, ivSt, nBD
5.	F/19	Asthma and allergic rhinitis	Immunotherapy with HDM mix (10 IR/ml 0.2 cc)	Wheezing	150/90	ivAH, imE, nBD
6.	F/39	Asthma and allergic rhinitis	Immunotherapy with HDM mix (10 IR/ml 1 cc)	Papular urticarial lesions	150/90	ivAH, ivSt, nBD, ACEi
7.	F/29	Asthma and allergic rhinitis	Immunotherapy with Phostal 350 (1 ml)	Papular urticarial lesions	160/100	ivAH, ivSt, nBD, ACEi
8.	F/23	Multiple drug, food and latex allergy, chronic urticaria, angioedema	Omalizumab treatment (300 mg/month)	Difficulty in breathing, tachycardia	150/100	ivAH, scE, imE, nE, ACEi

*M* male, *F* female, *ABP* arterial blood pressure, *HDM* house dust mite, *ivAH* IV antihistaminic, *ivSt* IV steroid, *scE* Subcutaneus Epinephrine, *imE* IM Epinephrine, *nBD* nebulized bronchodilator, *nE* nebulized epinephrine, *ACEi* angiotensin converting enzyme inhibitor

^a^Classical initial anaphylactic symptoms (generalized itching, flushing, swelling of the lips and skin, urticaria) are not mentioned here

^b^All patients basal blood pressures were normotensive

Following the HA reaction, and just before the initiation of anaphylaxis treatment, the systolic and diastolic BPs were measured, and were expected to be between 150 and 220 mmHg versus 90 and 150 mmHg, respectively, to be considered as HA. During treatment, 2 patients received epinephrine injections (one had additional nebulized epinephrine) without any complication. The BPs of patients who had received epinephrine were 150/90 and 150/100 mmHg, respectively. The symptoms of all patients recovered within minutes (1–10 min) following treatments.

We performed a 4-day-rush protocol on 2 of our patients (patients numbered 1 and 2) who had developed an anaphylaxis due to venom immunotherapy. One of these patients had developed a monthly maintenance level, while the other one had an initial build-up phase anaphylaxis. One patient who had received an immunotherapy due to HDM had a monthly maintenance level, whereas two patients had developed an initial build-up phase anaphylaxis (patients numbered 4, 5 and 6). Three of the patients who had anaphylaxis during the immunotherapy had also developed papular urticarial lesions during reaction (patients numbered 1, 6, and 7).

One patient (patient numbered 8) who had been diagnosed with multiple drug, food and latex allergy and receiving omalizumab injections for chronic urticaria and angioedema had anaphylactic reaction in 2nd year of treatment. Upon the start of this patient’s complaints, 0.5 mg of subcutaneous epinephrine and nebulized epinephrine was administered. Following this procedure, arterial BP was increased to 180/100 from 150/100 mmHg. Since the increased complaints indicating an upper airway obstruction, intramuscular 0.3 mg epinephrine was also administered. Then, the complaints resolved and high BP was normalized. The patient had received this treatment several times without experiencing any allergic reaction until the occurrence of anaphylaxis.

One patient (patient numbered 3) who was thought to have multiple food allergies, developed HA during fresh prick testing (prick to prick). This patient had a medical history of anaphylactic reactions (common body rash and erythema, swelling of the lips, dyspnea) following food intake a couple of times. No reaction was observed in the skin prick test performed commercially along with the ready-made food allergy test panel. On follow-up, this patient revealed that he had been experiencing new allergic reactions through various fruits which he had once eaten without a problem. He stated that he was having common rash and erythema on his body along with dyspnea after he ate apricot, plums and cherries at different times. He also stated that these complaints were accompanied by a blackout, palpitation and sort of a coma after he ate peach. A fresh prick testing was performed on the patient by administration of an apricot, a peach and a plum. An edema reaction was observed which was accompanied by erythemas with the sizes of 7 × 8 mm, 5 × 6 mm, 6 × 6 mm after peach, apricot and plum intake, respectively. Following this test, a severe anaphylaxis had developed within 10 min. He was then relieved with the administration of antihistaminic, steroid and nebulized bronchodilator therapy.

In patients with hypertensive anaphylactic reaction, the blood pressure returned to normal values with regression of the other symptoms. Despite the observed improvement, the patients were kept under observation and vital signs were monitored hourly for 6 h.

## Discussion

Anaphylaxis has an acute and unexpected onset; it may vary in severity and may resolve spontaneously. The clinical manifestations of anaphylaxis depend on the organ systems involved. Onset of symptoms, clinical severity, and sequence of symptom progression varies between individuals, and reactions may even vary in the same individual between episodes (Järvinen and Celestin 2014). Widely accepted criteria to help clinicians identify possible anaphylaxis emphasize the rapid onset of its multiple symptoms and signs (Sampson et al. 2006). Reduced blood pressure after exposure to an allergen (for adults: systolic blood pressure of <90 mmHg or >30 % decrease from that individual’s baseline) is the leading indicator of an anaphylactic reaction (Muraro et al. 2014). However, our results revealed that the prevalence of HA may be at a considerable rate (12.90 %), thus, hypotension may not be a standard symptom of anaphylactic reaction.

Among other symptoms of anaphylaxis, cutaneous manifestations occur in most cases, but it may develop without skin changes (Muraro et al. 2014). Respiratory or cardiovascular symptoms are the potentially life-threatening features of anaphylaxis (Simons et al. 2012). Respiratory symptoms occur more frequently in children, and cardiovascular symptoms predominate in adults (Muraro et al. 2014). In our cases, respiratory and cardiovascular symptoms were more prominent, which may be probably due to earlier ages of the patients.

During an anaphylactic reaction, immediate release of a series of mediators, including histamines, leukotrienes, prostaglandins, thromboxanes, and bradykinins from basophils and mast cells cause increased mucous membrane secretions, increased capillary permeability and leak, markedly reduced smooth muscle tone in blood vessels (vasodilation) and bronchioles, and bronchial smooth muscle contractions (Ecc Committee S, Task Forces of the American Heart A 2005). Internal compensatory vasopressor responses such as secretion of catecholamines, activation of angiotensin system, production of potent vasoconstrictor peptide endothelin-1 may induce unsteady reactions and in some patients, peripheral resistance can increase to abnormally high levels during anaphylactic episodes. Serotonin has an important role in the mechanism of anaphylaxis which may also lead to systemic hypertension over the receptors (serotonin I and serotonin II) (Isbister and Buckley 2005; Watts 2005).

Epinephrine is a sympathomimetic agent which has an effect on vasoconstriction, and bronchodilation. It also reduces mucosal edema. Epinephrine down-regulates histamine released from mast-cells and other mediators of inflammation. It is an optional drug for the treatment of anaphylaxis and should be injected promptly in the event of an anaphylactic reaction or when progression to anaphylaxis is likely to occur (Järvinen and Celestin 2014).

The differential diagnosis of anaphylaxis includes several medical diseases and somatoform disorders, which affect the organ systems most frequently involved in anaphylaxis (Muraro et al. 2014). Patients having anxiety and/or panic disorder had already been excluded from this study. No psychiatric pathology had been observed in study patients. All of our patients, except for one who had immunotherapy, had received their injections several times without experiencing any allergic reaction before the occurrence of a hypertensive anaphylactic reaction.

Allergen immunotherapy has been shown to be an effective treatment for respiratory allergies and hymenoptera venom allergy. The major risk of an immunotherapy, especially with the venom, is anaphylaxis, with an incidence of up to 12 % for hymenoptera venom (Lockey et al. 1990). It is expected to have more adverse effects on ultra-rush (few hours) compared to a rush protocol, and with rush compared to cluster protocols. In general, although there are commercial preparations used for skin tests, in the event that no commercial preparations containing the suspected allergen can be found or that the level of the allergen is thought to be quite low, it is possible to directly perform skin testings by diluting the food as it is consumed or through its juice (prick to prick test), and in this case, allergic reactions due to the non-standardized allergen may rarely occur (Bernstein et al. 2008). The risk of generalized allergic reactions to skin prick testing may be higher in poly-sensitized individuals and in those who underwent skin prick testing with multiple fresh foods.

Anaphylactic reactions have been associated with monoclonal antibodies and biological modifiers and risk of anaphylaxis with omalizumab is reported to be 0.2 % (Järvinen and Celestin 2014). In our study, the risk of anaphylaxis development with the administration of omalizumab was found to be 0.21 %, and that was a HA. In the literature, a severe anaphylaxis with hypertension after applying oxaliplatin (alkylating chemotherapeutic agent) was reported in two cases (Lee et al. 2007).

We assume that in minority of patients, early compensatory vasopressor response may be dominant, which can cause anaphylactic reactions manifesting with hypertensive attacks. Especially, in young patients, the possibility of a hypertensive attack in anaphylactic reactions should be kept in mind, and the arterial blood pressure should be measured prior to the epinephrine injection to avoid a potential dangerous side effect of epinephrine (Wendt et al. 2011). There is no information in the literature regarding rates of hypertensive anaphylactic reactions. We think that this is due to insufficient evidence of hypertension during the course of an acute anaphylactic reaction which is also perplexed by prompt administration of epinephrine in order to prevent potential life-threatening reactions like upper airway obstruction.

In conclusion, even though a hypertensive attack may occur in few of anaphylaxis patients, there must be no hesitation for using epinephrine during the potential life-threatening manifestations of anaphylaxis, such as upper airway obstruction. Anaphylaxis and hypertension can be recovered by epinephrine injection when required. According to the best of our knowledge, this study is the first original study about the prevalence of HA in English-medical literature.

## Abbreviations

BP: blood pressure
EAACI: the European Academy of Allergy and Clinical Immunology
GATA: Gulhane Military Medical Academy
HA: hypertensive anaphylaxis
IT: immunotherapy
HDM: house dust mite
